# Spontaneous blastocyst collapse as an embryo marker of low pregnancy outcome: A Time-Lapse study

**DOI:** 10.5935/1518-0557.20190044

**Published:** 2020

**Authors:** Romualdo Sciorio, K. J. Thong, Susan J. Pickering

**Affiliations:** 1 Division of Reproductive Endocrinology and Infertility, Garcia de Orta Hospital, Almada, Portugal

**Keywords:** embryo culture, embryoscope time-lapse monitoring, spontaneous blastocyst collapse, single blastocyst transfer, pregnancy outcome

## Abstract

**Objective:**

In this study we investigate the correlation between spontaneous blastocyst collapse and pregnancy outcome.

**Methods:**

This is a retrospective study performed at Edinburgh Assisted Conception Programme, EFREC, Royal Infirmary of Edinburgh, UK. Embryos were cultured individually in 6.0% CO_2_, 5.0% O_2_, 89.0% N_2_, using single step medium (GTL™ Vitrolife, Göteborg, Sweden) and selected for transfer using standard morphological criteria. Using the EmbryoScope™ time-lapse monitoring (TLM), blastocysts collapse was analyzed by measuring the maximum volume reduction and defined as having collapsed if there was >50% volume reduction. Couples undergoing IVF/ICSI treatment and having an elective single embryo transfer (eSET) at blastocyst stage were included in this study. After the embryo transfer, retrospectively, each blastocyst was allocated to one of two groups (collapsed or not collapsed). 62 blastocysts collapsed once or more during development (17.4%), the remaining 294 showed no collapse (82.6%).

**Results:**

A significantly higher implantation rate (IR) of 61.2% and ongoing pregnancy rate (OPR) of 53.7% was observed when blastocysts which had not collapsed were replaced compared to cycles in which collapsed blastocysts were replaced (IR rate 22.6% and OPR 17.7%).

**Conclusion:**

This study demonstrated that human blastocysts which collapse spontaneously during *in vitro* development are less likely to implant and generate a pregnancy compared with embryos which do not. Although this is a retrospective study, the results establish the utility of collapse episodes as new marker of embryo selection following eSET at blastocyst stage.

## INTRODUCTION

Over the past forty years assisted reproductive technologies (ART) have evolved considerably from and ambitious and experimental procedure to a mainstream medicine which has brought more than 7 million children to the world (De Geyter *et al*., 2018). Despite these improvements, live birth after ART treatment is not guaranteed, with almost half of the patients treated having an unsuccessful outcome, even after several cycles (Ghazal & Patrizio, 2017). However, due to the inefficiency of embryo implantation, multiple embryos are often transferred to the uterus at once, leading to higher rates of multiple gestations. In that respect, TLM plays an important role in embryo selection, by providing images and continuous assessment of human embryos, with minimal disturbance to the culture (Aparicio-Ruiz *et al*., 2016; Kirkegaard *et al*., 2012; Meseguer *et al*., 2011; Campbell, 2014; Basile *et al*., 2014).

Extending embryo culture to the blastocyst stage has become a routine in many IVF laboratories. The most widely used grading system is that originally proposed by Gardner & Schoolcraft (1999). Although the system does not cover all aspects of blastocyst morphology it has been very effective in classifying the appearance and compactness of the inner cell mass (ICM), the cohesiveness and number of trophectoderm cells (TE) and degree of expansion of the blastocoel cavity. Blastocoel formation starts through an initial secretion between the morula cells: the small cavity is then maintained and increased by actions of the membrane channels such as the Na^+^/K^+^-ATPase enzyme, which raises the salt concentration within the embryo, attracting water through osmosis (Watson *et al*., 2004). The increased water pressure gradually increments the size of the cavity which continues throughout the blastocyst formation (Baltz *et al*., 1997). At the same time, TE cells secrete lysine that is involved in zona pellucida (ZP) thinning and hatching. ZP thinning occurs in mammalian blastocyst prior hatching (Biggers, 1998). Blastocyst expansion, as well as ZP thinning and hatching *in vitro* have been described by TLM in several mammals (Gonzales *et al*., 1996; Glass *et al*., 1973; Gonzales & Bavister, 1995; Seshagiri *et al*., 2009).

However, in humans the hatching process is still not completed understood. Weak contraction and embryo pulsation may be beneficial to the process, although strong collapse, with a volume reduction >50% may negatively affect the hatching (Gonzales *et al*., 1996; Niimura, 2003). Blastocyst collapse was first described in mammalian embryos (Lewis & Gregory, 1929). They monitored *in vitro* cultured rabbit blastocyst for eight days by time-lapse cinematography, and found that blastocysts repeatedly contracted and re-expanded during their development. In humans, a recent study showed that some kinetic parameters such as time to reach the morula stage and blastulation were significantly shorter in blastocysts that displayed a collapse episode. Moreover, results reported a reduction of implantation rate from 48.5% to 35.1% if blastocysts that displayed a collapse episode were transferred (Marcos *et al*., 2015). The authors recommended against transferring such blastocysts if any other alternative is available.

The goal of the current study was to monitor the blastocyst collapse *in vitro* using TLM in order to provide more evidence between collapse event and pregnancy outcome for unselected IVF/ICSI patients. This information may be useful to better understand blastocyst expansion and hatching and may suggest new approaches for embryo selection, in order to improve pregnancy outcome after ART.

## MATERIALS AND METHODS

This is a retrospective consecutive cohort study including 356 IVF/ICSI cycles carried out at Edinburgh Assisted Conception Programme, Royal Infirmary of Edinburgh, between January 2016 and July 2018. All cycles were with patient’s own oocytes and fresh eSET at blastocyst stage. *In vitro* embryo culture was performed using EmbryoScope™ time-lapse monitoring (TLM) incubator (Vitrolife, Sweden).

For the purposes of this study, the blastocyst volume reduction was automatically measured using EmbryoViewer™ drawing tools and we have defined collapse if there was a volume reduction >50%, from the expanded blastocyst and the collapse event. If the volume reduction was <50% the event was considered as a contraction only. Blastocyst transfer was scheduled on the morning of day-5, approximately 116 to 120 hours after insemination. In this study we included only eSET at blastocyst stage, and embryo implantation was confirmed by an ultrasound scan for gestational sac with fetal heartbeat after seven weeks of pregnancy. The retrospective observational study design did not require ethical approval for the use of human subjects.

### Ovarian Stimulation, Egg Retrieval, Fertilization, Embryo culture and scoring

A detail of ovarian stimulation and egg retrieval has been previously described by Sciorio *et al*. (2018). The Cumulus-oocyte-complexes (COCs) were isolated from follicular fluid and then rinsed and cultured in 0.5ml equilibrated G-IVF™ medium (Vitrolife, Sweden) containing Human Serum Albumin (HAS, Vitrolife, Sweden), and then incubated at 37ºC in 6.0% CO_2_ in atmospheric air using the Hera cell 240 incubator (Thermo Scientific). Sperm used for either routine IVF insemination or ICSI procedure was collected by masturbation and processed using a standard method as described by Bourne *et al*. (2004). All oocytes were cultured in G-IVF™ (Vitrolife, Sweden) on the day of insemination (day-0), which was performed by IVF or ICSI according to the patient’s aetiology and history.

For IVF procedures, oocytes were exposed to 150,000 motile sperm/ml, approximately 38-42 hours post hCG injection. For ICSI cycles, COCs were exposed to 80 IU/mL of hyaluronidase (HYASE-10×™, Vitrolife, Sweden) and mechanically removed by gentle pipetting in buffered medium (G-MOPS™PLUS Vitrolife, Sweden) using denudation pipettes of progressively smaller diameter (Vitrolife, Sweden). MII oocytes were incubated at 37ºC in 6.0% CO_2_ in atmospheric air in G-IVF™ medium until ICSI was performed. ICSI was carried out using G-IVF™ medium, polyvinylpyrrolidone (ICSI™, Vitrolife, Sweden) and a Narishigue (Narishigue Group, Japan) or Research Instruments (Research Instruments Ltd, Cornwall, UK) micromanipulator system at 38-42 hours post hCG injection. Only MII oocytes were injected. After ICSI, oocytes were placed in independent drops of 10µl of culture medium (GTL™ Vitrolife, Sweden) covered with mineral oil (OVOIL™, Vitrolife, Sweden) and incubated at 37ºC in 6.0% CO_2_.

Fertilization was identified by the presence of two pronuclei approximately 16-19 hours after insemination or microinjection. At this stage, normally fertilized pronuclear stage embryos were allocated to the EmbryoScope time-lapse incubator for culture in a 12-well EmbryoSlide™, which is a specifically designed dish for the EmbryoScope™ imaging system (Vitrolife, Sweden). This slide has 12 individual wells for embryo culture, each well containing 20µl of culture media with 1.4 mls overlay of mineral oil to prevent evaporation. The *in vitro* culture was performed in an atmosphere of 6.0% CO_2_, 5.0% O_2_ and 89.0% N_2_ at 37ºC in single step medium (GTL™ Vitrolife) which is designed for TLM allowing undisturbed culture.

In the EmbryoScope embryo culture, images were acquired every 10 minutes in 7 focal planes and morphological assessment was made by examining a video of development using the associated EmbryoViewer™ software, without moving embryos from the incubation. Embryos were assessed morphologically at cleavage stage according to British Fertility Society and Association of Clinical Embryologists guidelines, published by Cutting *et al*. (2008). Blastocysts were classified according to degree of expansion of the blastocoel cavity (1-6), quality and cohesiveness of the inner cell mass and trophectoderm cells (A-C), using a Gardner’s score (Gardner & Schoolcraft, 1999). In order to avoid any major bias in the study, top quality blastocyst, based on morphology only, was selected for embryo transfer in both groups (collapse and not collapse). Elective single embryo transfer was performed at blastocyst stage on day-5 using transfer medium containing hyaluronan and recombinant human albumin (EmbryoGlue™, Vitrolife, Sweden).

### Definition of the blastocyst collapse

**Figure 1:**
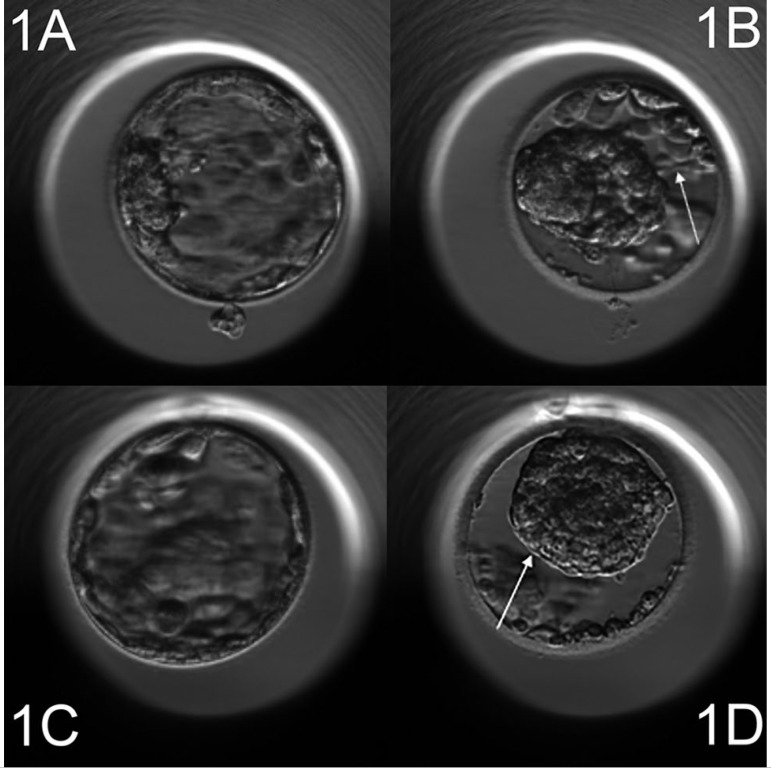
1a-1b, 1c-1d: Time-lapse monitoring of cultured human blastocyst. Arrow shows a collapse event (volume reductions more than 50%).

**Figure 2:**
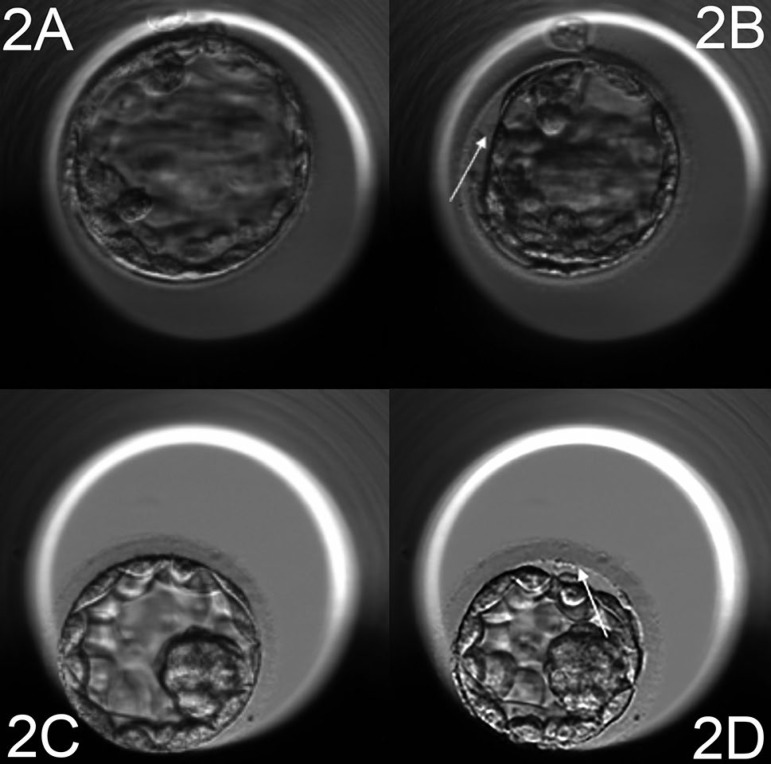
2a-2b, 2c-2d: Time-lapse monitoring of cultured human blastocyst. Arrow shows a contraction episode (volume reductions less than 50%).

To evaluate the collapse event, we used the EmbryoViewer™ workstation drawing tools in order to automatically measure the entire surface occupied by an embryo. Two measurements were performed: the first when the blastocyst was at the maximum expansion and the second, if the blastocyst showed a collapse episode, was carried out when the embryo occupied the smallest surface. Therefore, according to the maximum volume reduction we described two distinct episodes: “collapse” when the volume reduction was 50% or more (Figure 1: 1a-1b, 1c-1d). If there was an embryo pulsation with a volume reduction < 50% (as shown in Figure 2: 2a-2b, 2c-2d), this was considered as a “contraction” only.

### Pregnancy test and Clinical outcome

Chemical pregnancy was assessed based on serum βHCG levels 9 days after the embryo transfer. At seven weeks, a scan was performed to confirm the presence of fetal heart activity or gestational sac formation. The implantation rate (IR) was defined as the number of gestational sacs at the seven-week scan. A clinical ongoing pregnancy rate (OPR) was defined as a pregnancy with a fetal heart.

### Statistical Analysis

A Chi-square test was performed to examine the pregnancy outcomes between blastocyst which had a collapse event during the development and those which not. Differences were considered statistically significance at the level of *p*<0.05.

## RESULTS

A total of 356 consecutive cycles was analyzed and included in this study, all of which were eSET at blastocyst stage. A total of 62 blastocysts presented collapse episode (17.4%), of these twelve presented two, and one blastocyst presented three collapse events. The remaining 294 either contracted minimally or show no sign of collapse at any time (82.6%). There were no differences in the characteristics of patients who had a collapsed blastocyst transferred compared to those that had a transfer of a non-collapsed blastocyst. None of the parameters, including age, body mass index (BMI), year of infertility, days of stimulation, gonadotropin used and endometrial thickness were significant (*p*>0.05; Table 1).

**Table 1:** Clinical characteristic of patients which received a transfer of blastocyst which had a collapse event compared to those that did not

	Not collapsed (294 patients)	Collapsed (62 patients)	*p*
Age (years)	32.68±3.20	32.96±3.32	>0.05
BMI (kg/m^2^)	22.37±2.28	22.22±2.18	>0.05
Infertility (years)	5.01±3.04	5.16±3.20	>0.05
Stimulation (days)	11.46±1.10	11.31±1.22	>0.05
Gonadotropin used (IU)	2274.16±714.22	2231.52±748.52	>0.05
Endometrial Thickness (mm)	10.26±1.40	10.21±1.48	>0.05

BMI: body mass index, IU: international unit.

Summarized in Table 2 are reported the baseline cycle characteristics in groups where collapse episode were observed or not. The total number of oocyte collected, fertilized and blastocyst formation was comparable in both groups (*p*>0.05; Table 2).

**Table 2:** Baseline characteristic of the patients which received a transfer of blastocyst which had a collapse event during the development compared to those that did not. There was no statistical significant difference among laboratory parameters such as oocyte number, fertilization, good quality blastocyst or blastocyst utilization (2PN: two pronuclei, OPU: Oocyte Pick Up).

	Not collapsed (294 patients)	Collapsed (62 patients)	*p*
Age (years)	32.68±3.20	32.96±3.32	>0.05
Mean number oocytes collected	12.18±2.88	12.41±3.18	>0.05
Number of Mature Oocytes	10.09±2.66	10.209±2.98	>0.05
Normal fertilisation rate (2PN/Oocyte collected)	67.44%	66.98%	>0.05
Mean number 2PNs per cycle	7.98±2.58	7.87±2.87	>0.05
Good Quality Blastocyst	3.12±2.25	3.08±2.23	>0.05
Blastocyst Utilization Rate (Blast/2PN)	62.68%	61.88%	>0.05

Blastocysts were classified according to degree of expansion of the blastocoel cavity and quality of the inner cell mass and trophectoderm cells. On the morning of day-5, the top quality blastocyst was selected for transfer by considering the morphology only, regardless the collapse episode. Following embryo replacement, the analysis was performed and each blastocyst was allocated to one of two groups (collapsed or not collapsed). No difference has been noted on blastocyst collapse event in relation to the insemination technique adopted [32/62 (51.6%) in IVF *versus* 30/62 (48.4%) in ICSI].

The results described in Table 3 compare clinical outcomes between patients who had transfer of a collapsed blastocyst *versus* a non-collapsed blastocyst. A significantly higher positive pregnancy test rate of 69.4% was observed when blastocysts which had not collapsed during development were replaced compared to cycles in which collapsed blastocyst were transferred 40.3%. In addition, a significantly higher ongoing pregnancy rate of 53.7% was noticed when blastocysts without any collapse were transferred compared to blastocysts which exhibited a collapse event during their development 17.7%. The miscarriage rate was higher in the collapsed group 24.2% *versus* non-collapsed 15.6% but this trend was not statistically significant (Table 3).

**Table 3:** Pregnancy outcome of the patients which received a transfer of blastocyst which had a collapse event during the development compared to those that did not

	Not collapsed (294 patients)	Collapsed (62 patients)	*p*
Positive Beta (%)	204/294 (69.4)	25/62 (40.3)	<0.0001
Imp. Rate (%)	180 /294 (61.2)	14/62 (22.6)	<0.0001
Ongoing P. Rate (%)	158/294 (53.7)	11/62 (17.7)	<0.0001
Miscarriage Rate (%)	46/294 (15.6)	15/62 (24.2)	>0.05

## DISCUSSION

Previous studies in humans have already demonstrated that during *in vitro* embryo culture, blastocyst collapse may be adversely linked to pregnancy outcome (Marcos *et al*., 2015; Bodri *et al*., 2016). Accordingly, the aim of this study was to assess whether blastocyst collapse could be a predictive negative marker for embryo implantation. *In vitro* blastocyst collapse and re-expansion is still not clear. Niimura (2003) described in a mouse model that blastocyst showing consecutive weak contractions reached, mostly, the stage of hatching, while those suffering strong collapse/s failed to hatch. In addition, with the use of electron microscopy, the authors described the formation of intercellular ridges between trophectoderm cells on the surface of expanded blastocysts. Interestingly, after the collapse event such intercellular ridges disappear. Similar results were also reported by Iwata *et al*. (2014), they observed that some human blastocysts undergo several collapses episodes and then finally degenerate. The number of collapses was significantly higher in unhatched blastocysts compared to those which hatched successfully. A study published by Togashi *et al*. (2015) investigated the role of gap junctions in *in vitro* mouse embryo development by TLM. Particularly, they examined the role of gap junction inhibitors such as oleamide and heptanol. Severe collapse events were significantly more frequent in embryos cultured with oleamide or heptanol compared to the control group. The authors suggested that inhibition of gap junctions could delay growth to the blastocyst stage and induce collapse events. Gap junctions are fundamentally important not only for intracellular communication, but also in the maintenance of cellular homeostasis (Houghton, 2005). In line with that, although we cannot directly demonstrate that dysfunction of gap junctions is the cause of the collapse episode observed, however, we hypothesize that there may be a relation between these phenomena and that disruption of gap junctions may negatively affect pregnancy outcomes.

In the current study, we used a value of 50% for blastocyst collapse for the following reason: an obvious collapse event (>50% volume reduction) could be clearly recognized, additionally, if any reproductive effect is caused by this behaviour it could be more perceptible. Our data are in agreement with a study presented by Harton *et al*. (2016) at the ESHRE, the authors examined the relationship between blastocyst collapse patterns and euploid human embryos resulting in live birth. Results showed euploid blastocysts that resulted in live birth had statistically significantly lower frequency of a collapse episode. Therefore, blastocysts that experienced collapse were less likely to achieve live birth.

In the present study we observed that a small number of blastocyst presented multi-collapsed events (twelve blastocysts had two collapses and one blastocyst three). Due to the small number of those embryos we did not perform any analysis comparing clinical outcome between embryo showing one collapse and those showing multiple collapses. The same was also related to blastocysts which show a weak contraction (<50%), this was not considered as a collapse event. The results presented in this study were only related to the two groups analyzed (collapse and not-collapse).

Another aspect analyzed was the relationship between the fertilization method and the collapse episode. Since at the ICSI time an injection needle needs an opening through the ZP, this manipulation might have an influence on the collapse episode. Indeed the analysis performed has shown no difference in the blastocyst collapse event in relation to the insemination technique used (51.6% in IVF *versus* 48.4% in ICSI).

Blastocyst expansion is of considerable value in embryo selection. Ahlström *et al*. (2011) reported that trophectoderm morphology and expansion were the best predictors of live births in over 1000 single blastocyst transfers. Van den Abbeel *et al*. (2013) found that blastocyst expansion was the most important parameter predicting pregnancy outcome and live birth. Perhaps, this reflects a benefit to robust cellularization and epithelial cohesiveness (Kovacic *et al*., 2004; Thompson *et al*., 2013). The expansion is dependent on several mechanisms including compaction between polarized blastomeres, construction of junctional complexes and deployment of ion and water transporting systems driving the formation of the fluid-filled blastocoel cavity (Baltz *et al*., 1997; Ducibella *et al*., 1975; Bell & Watson, 2013; Biggers *et al*., 1988; Madan *et al*., 2007; Marikawa *&* Alarcon, 2012). When blastocyst development is accompanied by severe collapse and re-expansion event(s), hatching does not occur even if the zona is already broken (Massip *et al*., 1982; Massip & Mulnard, 1980) and this may be associated with a functional and structural anomaly of the trophectoderm cells. In addition, the molecular mechanisms for blastocyst collapse could be also related to physiological stress and/or excessive energy consumption. The re-expansion process after a collapsed episode requires a lot of energy, and occurs by active transport of Na^+^ into the blastocoelic cavity. Study performed on mouse model indicates that the demand for Na/K-ATPase raised after strong collapse occurred (Niimura, 2003). Renard *et al*. (1980) reported differences in the metabolic activity, particularly concerning glucose uptake, between bovine blastocysts. In particular they found that blastocyst which failed to expand after 20 hours culture period, did not take up glucose from the medium. Since glucose is a necessary factor for *in vitro* blastocyst development, it has been hypothesized by Wordinger & Brinster (1976) that strong collapse might detrimentally influence the metabolism and embryo viability.

Although in the present study we did not perform any pre-implantation genetic screening, the chromosomal abnormalities could perhaps clarify the difference in clinical outcomes between the two groups (collapse and not collapse). A recent study published by Viñals Gonzalez *et al*. (2018) investigates the correlation between blastocyst collapse patterns and genetic complements in human embryos. Eight hundred ninety-six good quality blastocysts were analyzed. Results showed a statistical difference in the number of collapse episode being reported greater in aneuploid when compared to euploid embryos. Moreover, in agreement with our study, a reduction in the pregnancy rate was reported if blastocysts that displayed a collapse episode were transferred, compared to the non-collapse group (47.6% *versus* 78.5%, respectively). Although this is the first study to report such data, it might be clear that spontaneously blastocyst collapse, despite being a physiological feature during blastulation, is conditioned by the ploidy status of the embryo (Viñals Gonzalez *et al*., 2018). In conclusion, the analysis of spontaneous blastocyst collapse could be used as tool to improve embryo selection, especially when there are several blastocysts available for transfer. Based on our findings, we suggest that observations of the collapse event at blastocyst stage should be used as an index for blastocyst evaluations, and could help to further improve IVF/ICSI outcome following eSET at blastocyst stage. It needs to be mention as limitation that this is a retrospective analysis and lack of randomization, therefore the conclusion need to be taken with caution. Finally, live birth rate should also be investigated to see if there is a difference in final outcome between the two groups.

### Compliance with ethical standards

**Human and animal rights**. All procedures performed in studies involving human participants were in accordance with the ethical standards of the institutional and with the 1964 Helsinki declaration and its later amendments. For this type of study, formal consent is not required.
